# DECOMP Report: Answers surgeons expect from an abdominal wall imaging exam

**DOI:** 10.1590/0100-6991e-20223172en

**Published:** 2022-04-13

**Authors:** CHRISTIANO MARLO PAGGI CLAUS, MARCIO CAVALIEIRI, FLÁVIO MALCHER, CARLOS TRIPPIA, ANTONIO LUIS EIRAS-ARAUJO, ERIC PAULI, LEANDRO TOTTI CAVAZZOLA

**Affiliations:** 1 - Universidade Positivo, Clínica Cirúrgica e Cirurgia Minimamente Invasiva - Curitiba - PR - Brasil; 2 - Hospital Municipal Lourenço Jorge, Clínica Cirúrgica - Rio de Janeiro - RJ - Brasil; 3 - New York University Grossman School of Medicine, Abdominal Core Health - New York - NY - Estados Unidos; 4 - Hospital Nossa Senhora das Graças, Radiologia - Curitiba - PR - Brasil; 5 - Universidade Federal do Rio de Janeiro e Instituto D’Or de Ensino e Pesquisa, Radiologia - Rio de Janeiro - RJ - Brasil; Instituto D’Or de Ensino e Pesquisa, Rio de Janeiro, RJ, Brazil; 6 - Penn State Hershey Medical Center, Minimally Invasive and Bariatric Surgery - Hershey - PA - Estados Unidos; 7 - Universidade Federal do Rio Grande do Sul, Cirurgia - Porto Alegre - RS - Brasil

**Keywords:** Hernia, Hernia, Abdominal, Tomography, Radiology, Hérnia, Hérnia Abdominal, Tomografia, Radiologia

## Abstract

Abdominal wall (AW) hernias are a common problem faced by general surgeons. With an essentially clinical diagnosis, abdominal hernias have been considered a simple problem to be repaired. However, long-term follow-up of patients has shown disappointing results, both in terms of complications and recurrence. In this context, preoperative planning with control of comorbidities and full knowledge of the hernia and its anatomical relationships with the AW has gained increasing attention. Computed tomography (CT) appears to be the best option to determine the precise size and location of abdominal hernias, presence of rectus diastase and/or associated muscle atrophy, as well as the proportion of the hernia in relation to the AW itself. This information might help the surgeon to choose the best surgical technique (open vs MIS), positioning and fixation of the meshes, and eventual need for application of botulinum toxin, preoperative pneumoperitoneum or component separation techniques. Despite the relevance of the findings, they are rarely described in CT scans as radiologists are not used to report findings of the AW as well as to know what information is really needed. For these reasons, we gathered a group of surgeons and radiologists to establish which information about the AW is important in a CT. Finally, a structured report is proposed to facilitate the description of the findings and their interpretation.

## RESUMO

Hérnias da parede abdominal são um problema bastante comum enfrentado pelo cirurgiões gerais. De diagnóstico essencialmente clínico, as hérnias abdominais durante muito tempo têm sido consideradas um problema de simples reparo. Entretanto, o acompanhamento de longo prazo dos pacientes têm demonstrado resultados desapontadores, tanto em termos de complicações quanto risco de recidiva da hérnia. Neste contexto, o planejamento pré-operatório com controle de comorbidades e pleno conhecimento da hérnia e suas relações anatômicas com a parede abdominal têm ganho cada vez mais atenção. A tomografia de abdome parece ser a melhor opção para determinar o tamanho e localização precisos das hérnias abdominais, presença de diastase de músculo reto e/ou atrofia da parede associada, assim como proporção da hérnia em relação a parede abdominal. Essas informações podem auxiliar o cirurgião na escolha da melhor técnica cirúrgica (aberta vs. MIS), posicionamento e fixação das telas, e eventual necessidade de aplicação de toxina botulínica, pneumoperitônio pré-operatório ou técnicas de separação de componentes. Apesar da relevância dos achados, eles são raramente descritos em exames de tomografia uma vez que os radiologistas não estão acostumados a olhar para a parede abdominal assim como não sabem quais as informações são realmente necessárias. Por estes motivos, nós reunimos um grupo de cirurgiões e radiologistas visando estabelecer quais são as informações da parede abdominal mais importantes em um exame de tomografia assim como propor um laudo estruturado para facilitar a descrição dos achados e sua interpretação.

## TECHNICAL NOTE

For many years, the diagnosis and evaluation of hernias of the abdominal wall (AW) was made through physical examination and it was considered a waste to request an imaging study to evaluate “only” a hernia. The diagnosis is usually clinical, based on symptoms and physical examination. Increasingly, however, surgeons interested in hernia repair find utility in imaging exams^1^. They may be necessary to confirm hernia defect size, for example a hernia occurring in the setting of obesity or an incisional hernia with multiple defects under a fibrotic surgical scar. Imaging may help surgeons on surgical planning. The size and content of the hernia, the volume of the hernia sac, the hernia’s proximity to bone structures or even associated muscle atrophy can all be determined on imaging. These guide the need for preoperative measures or intra-operative measures to increase the compliance/volume of the abdominal cavity, define the approximate size, best position for mesh and mesh fixation methods and well as suggest both perioperative risks and postoperative results^2^ ^-^ ^4^.

Ultrasonography (US), computed tomography (CT) or magnetic resonance imaging (MRI) all have value to the hernia surgeon. The value increases when imaging is used in conjunction with a thorough history and physical examination. Nevertheless, the lack of uniform definition of what a hernia is and the lack of standardization in the interpretation of image examinations create difficulties in both clinical care and academic research; there is simply a paucity of high-level scientific evidence on hernia imaging^1^. For example, an incisional hernia may be defined as “a defect/hole, with or without bulging” in one study but as abdominal “wall weakness” in another^5^. Likewise, radiologists do not utilize standard reporting techniques when looking at the abdominal wall and commonly report clinically irrelevant hernia-related information while neglecting critically important information. Hernia surgeons and radiologists commonly have different interpretations of the same imaging study^6^ ^,^ ^7^. 

Despite reporting inaccuracies, most studies comparing the accuracy of diagnostic methods point to CT as the exam of choice^1^ ^,^ ^5^ ^,^ ^8^. It shows the best correlation between the imaging findings, physical examination, and the operative findings, in addition to allowing the images to be reassessed by the surgical team or other radiologists later. Notably, CT scans are free of the operator factor present in ultrasound acquisition. There is generally no need to use intravenous contrast. Although CT images are static, they can be performed with maneuvers that increase intra-abdominal pressure, which facilitates the identification of abdominal hernias^1^ ^,^ ^8^. 

Ultrasonography, in addition to being less expensive and widely available, is a more dynamic exam and can be very useful with the interaction of the patient, making it possible to perform maneuvers to increase intra-abdominal pressure and point out the site of pain or bulging^1.9^. Unfortunately, ultrassonography accuracy depends on the operator and the images generated are often of little value to the surgeon. Magnetic resonance imaging is more expensive, less available and there are no studies showing its superiority in relation to CT in the evaluation of abdominal wall defects^1^. In addition, the images generated are generally more difficult for surgeons to assess, since they are not used to interpreting them^5^.

The use of the imaging in the context of AW hernias goes far beyond just establishing confirmation of the diagnosis, but it allows the evaluation of complex (large) hernias, their content and the relationships between bone structures and adjacent muscles. This knowledge, combined with the emergence of new surgical techniques and a better understanding of the anatomy of the AW by surgeons, allows surgical planning in an individualized way. For this, the standardization of the necessary information for an image exam is essential, but unfortunately this is not yet routine. To overcome this issue, surgeons must interact more with the interpreting radiologist, or (alternatively) become experts in the proper evaluation of these imaging studies^4^ ^,^ ^10^.

To guide surgeons and radiologists, we created a study group, including representatives of the two specialties, aiming to create guidelines for surgeons and mainly radiologists on the fundamental descriptions for an adequate assessment of the AW from an image perspective. Information was grouped into four topics covering: defect; hernia content; musculature and previous event. In each section, we made a brief correlation of the aspects required for the exam with its relevance from a practical/clinical point of view. The ultimate goal is the proper planning of the surgery. To make reading more dynamic, we organize it in a Q&A format. At the end, we propose a structured report model aimed at standardizing relevant information: the DECOMP report (DEfect; COntent (hernia); Musculature; Previous events).

### Image information for evaluation of ventral hernia

Like any imaging exam, a good interpretation begins with a descriptive indication from the surgeon, for whom the radiologist can seek answers to the main questions about the evaluated case. Good communication between the requesting professionals and the executors is essential and the kickoff is a good and clear indication of the exam and expectations.

### General CT Protocol

Technical parameters of the examination do not differ from those when performing “regular” abdominal CT. The only points that deserve more attention are:

- the use of intravenous (IV) contrast is not essential for assessing the abdominal wall defects and can be avoided.
- in suspicion of incarceration / strangulation IV contrast may show signs of impairment of vascular supply to the hernia content like reduction or absence of parietal enhancement or even engorgement of the mesenteric vessels
- the same goes for the postoperative evaluation where IV contrast is not necessary, except in cases of collection with suspected infection where it can help to differentiate it.
- Valsalva maneuver is only necessary if there is diagnostic doubt. In cases where the diagnosis is already established, it usually does not provide additional information. Although some surgeons may consider it different, the diagnosis of loss of domain must be done during the examination without Valsalva.

## DEFECT

### 1. What is the defect location?

The precise location of the hernia defect is essential for the planning and execution of the surgery. Umbilical stalk, xiphoid and pubic symphysis should be used as a reference for midline hernias; and costal margin, iliac crest, semilunaris line and midline for lateral hernias.

In addition, hernias close to bony structures are usually considered more complex for repair. This is since the musculature close to bone structures has less complacency, as well the greater difficulty creating space for overlapping and mesh fixation. This information can alter the operative technique, planned mesh position and fixation method.

### 2. How big is the hernia defect?

**Figure 1:**
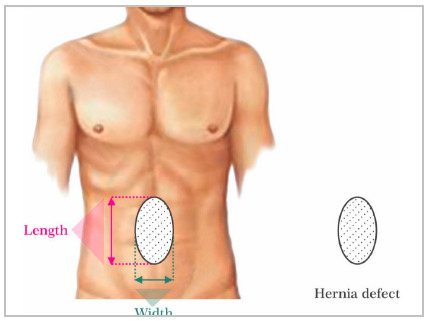
Schematic illustration of the measures of the hernia defect in two axes: longitudinal and transverse, according to the EHS classification.

A simple measurement of the size of the defect is one of the most important aspects for surgical planning. A linear measurement of the largest hernia dimensions in both longitudinal and transverses axis is required (Figure 1). This allows a classification of the hernia (small/medium/large) helping to predict the degree of difficulty of fascial closure. We suggest the use of the European Hernia Society (EHS) classification of primary and incisional AW hernias^11^. In this way, the surgeon can: 

- decide the surgical technique to be employed (conventional/open surgery vs laparoscopic/robotic). Very large or large hernias (diameter over 10cm) are less prone to minimally invasive techniques due to the technical difficulty of adequately closing the defect.
- indicate preoperative patient preparation measures such as the use of botulinum toxin chemodenervation and/or induced progressive pre-operative pnemoperitoneum, aiming to paralyze the lateral muscles and increase abdominal compliance, and allow reduced tension at defect/midline closure.
- planning for the need for component separation technique(s).
- define approximate mesh size and type. This is critically important in practice locations where the mesh needs to be requested and acquired preoperatively.

## 3. How many hernia defects are present?

**Figure 2:**
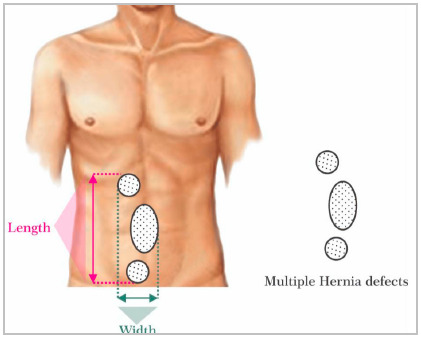
Schematic illustration for measuring multiple defects, according to EHS classification.

When there is more than one defect, information on the locations and the distance between the first and last defect edges. The same goes for incisional hernias with multiple defects (Figure 2). Additional and minor defects undiagnosed by physical examination may go unnoticed during surgery, especially if performed using open technique.

## HERNIA CONTENT

### 4. Does the hernia contain viscera?

Presence of viscera such as intestinal loops or bladder inside the hernia sac brings some important information about the case. First, it may indicate a greater risk of complications such as intestinal obstruction and greater severity in the event of a strangulation. The knowledge of this fact can help in defining the need for surgery or at least in planning the time when it should be performed. In addition, the presence of viscera inside the hernia sac is associated with greater difficulty in dissecting it and greater risk of an iatrogenic injury compared to hernias that contain only fat.

### 5. Is the hernia content incarcerated or strangulated?

Incarceration of the hernia content is an indication of greater surgical difficulty. The risk of complications, including possible iatrogenic injury to incarcerated content is greater. The surgeon may need maneuvers for reduction such as external compression, intra-operative enlargement of the hernia defect, among others. Incarceration, defined when the hernia content cannot be reduced manually, is generally of clinical diagnosis and cannot be given by imaging methods alone. Despite this, some of the aspects found may be indicative, increasing the suspicion of hernia incarceration:

- presence of hernia sac with narrow neck in a small defect in the wall;
- presence of fluid inside the hernia sac;
- parietal thickening (greater than or equal to 4mm) and/or distention of the herniated intestinal segment and dilation of the intestinal loops upstream inside the abdominal cavity (apart from the first criterion, the others are considered an imminent risk of strangulation of the incarcerated content).

Presence of these criteria, with incarcerated intestinal content, is a factor that should draw the attention of both the radiologist and the surgeon to a probable indication for urgent surgery.

**Figure 3:**
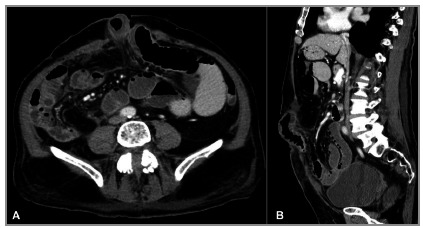
CT image showing a strangulated abdominal hernia: small bowel loop segment with signs of ischemia characterized by parietal thickening, mesentery densification, fluid in the hernia sac, and slight reduction in contrast uptake. In addition to distention of the proximal intestinal loops.

Strangulated hernia is defined when the vascular supply of the herniated content is compromised, determining ischemia. As image criteria that determine the suspicion of the diagnosis of strangulation of the hernia content we can observe (Figure 3):

- dilation of the segment of the intestinal loop located inside the hernia sac in the shape of a “U” or “C”, due to obstruction of the afferent and efferent intestinal segments (“closed loop” obstruction).
- thickening of the intestinal wall.
- hypo or hyperattenuation of the wall in relation to the usual pattern and parietal low uptake by means of intravenous contrast of the ischemic intestinal segment.
- intestinal pneumatosis.
- fluid inside the hernia sac.
- engorgement of the mesenteric vessels and obliteration of the mesenterial adipose plane of the herniated segment.

Presence of one or more of these findings is suggestive of strangulation of the hernia content and urgent surgical intervention should be considered^12^ ^,^ ^13^.

### 6. What is the relationship between sac/hernia content volume and abdominal cavity volume?

The relationship between the volume of the hernia sac versus the volume of the abdominal cavity is the best criterion for defining whether there is a loss of domain (LOD). LOD is defined when the volume of the sac/hernia content is greater than 25% of the volume of the abdominal cavity1^4^ ^,^ ^15^. This fundamental information allows the surgeon to consider that:

- there may not be enough space to reduce all herniated contents into the abdominal cavity and still achieve a complete primary fascial closure.
- the significant increase in intra-abdominal pressure, due to the reduction of a large volume of content in the abdominal cavity, can cause important ventilatory restriction due to the upward compression of the diaphragm. Although rare, patients may even develop abdominal compartment syndrome^16^. In addition, the risk of hernia recurrence and even evisceration are significantly increased^17^.

With this assessment prior to the procedure, surgeons can use several strategies to reduce the consequences of the condition described above. Although it is not the objective of this work, we can cite as alternatives: preoperative preparation (weight loss and respiratory physiotherapy); progressive preoperative pneumoperitoneum and application of botulinum toxin preoperatively; visceral-reduction surgery; component separation techniques. All these possibilities aim to increase abdominal capacity and/or compliance^18^ ^-^ ^21^.

**Figure 4:**
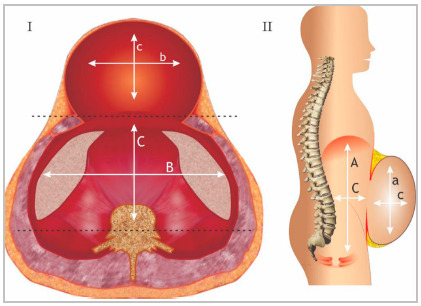
Representative illustration of the evaluation of the volume of the abdominal cavity and the volume of the hernia sac in suspected cases of LOD.

**Figure 5:**
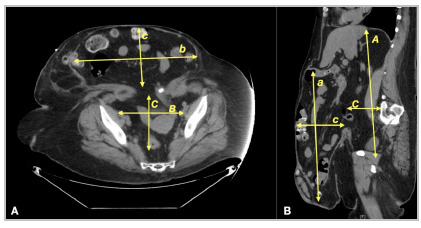
CT image illustrating how measurements are performed to assess the volume of the abdominal cavity and hernia sac in suspected cases of LOD.

For tomographic assessment of the risk of LOD in bulky hernias, we used the method described by Tanaka et al because it is simple to understand and can be performed by most mutislice tomographic equipment, as well as being easily measured by the radiologist at their standard workstation (Figures 4 and 5)^14^. The hernia sac and the abdominal cavity are considered ellipsoid structures, allowing the calculation of volume estimation using the multiplication between the measurements in a straight line of the longitudinal axes (cranio-caudal - CC, transversal - T and anteroposterior - AP) by the constant volume, according to the simplified formula:

Volume of the hernia sac or abdominal cavity: CC x T x AP x 0.52 

Some pre-established criteria are important for greater precision in calculating the volume:

A- The largest measurement of each axis should be used, even in different tomographic sections to calculate the volume of the hernia sac, as well as the volume of the abdominal cavity.
B- To determine the abdominal volume cavity (AVC), some reference points must be used: The measurement of the anteroposterior axis of the abdominal cavity is determined by the line that joins the muscle groups of the healthy (anterior) wall and the line that passes through the transverse processes of the vertebra (posterior). The CC distance is made between the first cut showing the diaphragm and the last cut showing the tailbone. The transverse distance (T) through the parietal peritoneum on each side of the abdominal cavity.
C- To determine the hernia sac volume (HSV), the following references should be used: Measurement of the limits of the parietal peritoneum of the hernia sac for the CC and T axes. For the AP axis, the distance between the anterior parietal sac peritoneum hernia to a line that joins the muscle groups of the healthy wall (posterior limit).

RV=HSV/AVC

if >25% = loss of domain

## MUSCULATURE

### 7. Does the patient have Rectus Abdominal Diastasis (RD)?

RD is defined as the increase in the distance between the medial edges of the two abdominals of the rectus abdominis muscle in the anterior midline, caused by laxity and thinning of the aponeurosis of the alba line^22^. The rectus abdominis muscles may have normal thickness or more often atrophy (myoaponeurotic laxity). This distance can be measured clinically by digital pulp or quantitatively by imaging methods such as US, CT or MRI. Although there is no consensus on the normality of the distance between the medial edges of the rectus abdominis belly, most of the literature considers the presence of diastasis when greater than 20mm. In addition, it can be classified according to the position (just above the umbilicus, only below the umbilicus, at the level of the umbilicus or even in complete)^23^ ^-^ ^25^.

**Figure 6:**
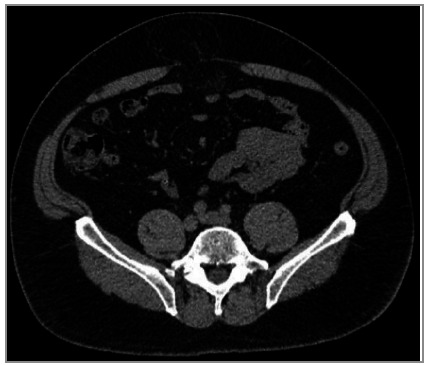
CT image showing a small hernia defect but with important diastasis/weakness of the AW.

RD is usually asymptomatic, and often not even noticed by patients. It does not cause complications and its treatment, from an aesthetic point of view, is usually addressed by plastic surgeons. However, RD represents a weakness of the midline abdominal wall^22^. Patients with midline hernias (umbilical, epigastric or incisional) with associated RD may be at increased risk of hernia recurrence or bulging in the postoperative period (Figure 6). Currently, important changes in the choice of surgical procedure have occurred in patients with midline hernias associated with RD. In general, more comprehensive surgeries or at least with mesh have been recommended to reduce the effects of weakness caused by RD on the risk of recurrence. Information regarding the presence or absence of RD (and the size of the longest distance / separation of the rectus abdominis muscles) should be part of every image evaluation report of the abdominal wall.

### 8. What is the width of the rectus muscles?

**Figure 7:**
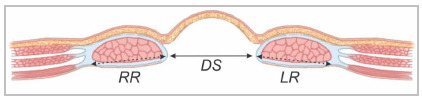
Schematic representation of the measurements of the hernia defect (DS) and right (RR) and left (RL) rectus muscle case according to the Carbonel equation.

**Figure 8:**
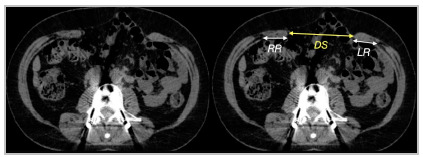
CT image showing the relationship between the measurement of the defect (DS) and the rectus muscles (RR and RL) to assess any need for separation of chemical or surgical components.

Although we understand that this information is a little more specific, the transverse measurement of the rectus abdominis width on each side (at the height of the largest transverse measurement of the defect) in relation to defect size is used as a predictor for the need for muscle component separation, as described in the Carbonel Equation^26^. Rectus defect ratio is calculated by simple addition of the right and left rectus widths divided by the hernia width (Figures 7 and 8). Authors reported that approximately 80% of patients with RDR <1 require components separation whereas when RDR was >2 only 10% required CS. Measuring the diameter of the rectus muscle is only necessary for midline hernia.

RDR=RR+LR/DS

RR - right rectus

LR - left rectus

DS - defect size

### 9. Is there muscle rupture or atrophy?

Patients who have already undergone any surgical intervention may have muscle weakness, atrophy (denervation) or muscle tissue loss. This usually occurs after surgeries with lateral incisions or flanks (lumbotomy type) that can cause damage to the neuro-vascular bundles that supply the central abdomen. Denervation atrophy of the musculature medial to the nerve transection may occur. In addition, denervation may result in bulging without a formal hernia defect (pseudo-hernia). It is important to differentiate between a hernia and a “pseudo-hernia” as denervation related bulging and asymmetry can persist after repair and can progress over time. Setting expectations in the pre-operative period is key^27^.

Breast reconstructions with transposition of the rectus abdominis muscle flaps (TRAM) after mastectomy are still common, and may lead to important weakness of the abdominal wall and bulging. All these findings certainly interfere not only in the planning of the surgical tactic but also in the postoperative functional and cosmetic results.

### 10. Is there an intraparietal hernia?

**Figure 9:**
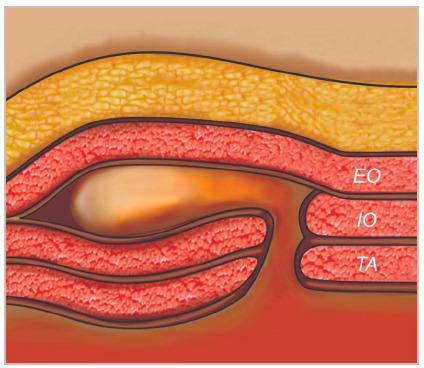
Illustration demonstrating intraparietal hernia. External oblique muscle is intact.

Some hernias, especially on the lateral wall of the abdomen, can occur without a defect of all muscle groups layers. In these cases, usually the external oblique muscle/aponeurosis is intact, and the hernia is contained within the muscular layers of the AW (Figure 9). Without this information, the surgeon may miss the hernia defect during an open approach, when they encounter the intact external oblique myofascial layer. 

## PREVIOUS EVENTS

### 11. Are there any signs of previous surgical manipulation or complication?

Presence of a mesh or even tacks for fixation may indicate previous surgical manipulation and therefore a greater degree of technical complexity in an upcoming repair. This may be even more relevant in cases where the mesh was placed in the intraperitoneal position. It is true that in some cases, neither the meshes nor the fixation devices (currently absorbable devices, radiotransparent, are becoming more popular) are detectable, but indirect signs such as loss of contour of anatomical planes can indicate them. 

The importance of the detailed description of the surgeon regarding possible repairs or previous interventions in the indication of the exam is crucial to allow the interpretation of subtle findings by the radiologist. A comprehensive review of the patient’s prior operative reports can indicate the type of mesh, its location and anatomic plane of implantation, which can make identification possible. Additionally, looking for non-anatomical folds and wrinkles from mesh contracture can facilitate mesh identification on CT imaging. 

Evaluation of collections or fistulous tracts (sinuses) by imaging is also important to decide on surgical steps such as dividing the treatment into steps to resolve the entire chronic infection or foreign body reaction before a new attempt at definitive repair with a new prosthesis.

The concept of a standardized report for abdominal wall imaging exams is proposed here:

## DECOMP report

### DEFECT

1- Location of the hernia defect(s):

A) Midline: cm from the xiphoid and/or pubic symphysis and/or the umbilical scar

B) Lateral: which anatomical position? cm from iliac crest and/or costal margin and/or linea alba / linea semilunaris

2-Defect size (measured on the transverse and longitudinal axes, respectively: cm).

3- Presence of more than one hernia defect:

- Not.

- Yes (distance between the defect ends: -cm) - repetition of the entire description of the second hernia defect according to the structured report above.

### CONTENT

4- Incarceration and/or strangulation signs:

- Not

- Yes: what signs:

5- Content of the hernia sac:

Adipose tissue / omentum / intestinal loop (which segment) / bladder / ureter / vascular structures (which) / solid organ/ other

6- Relationship between the volume of the hernia sac in relation to the volume of the abdominal cavity (>25%):

- Not

- Yes: risk of loss of domain

### MUSCULATURE

7- Rectus Muscles diastasis:

Not

Yes: width (mm) / extensions (cm) / position

8- Transverse width of the rectus muscles (just for midline defects):

- width:right: mm

- left: mm

9- Presence of atrophy and/or denervation of the AW muscles

- Not

-Yes: location / muscle group(s) / thickness (mm)

10- The hernia is intraparietal?

- Not

- Yes: location

### PREVIOUS EVENTS

11- Signs of previous manipulation, mesh implant or complication in the cavity and/or abdominal wall:

- Not

- Yes: which / where

## CONCLUSIONS

The better understanding between surgeons and radiologists in the evaluation of hernia of the AW will likely improve hernia repair outcomes.

We understand that this proposal brings more challenges to radiologists during the evaluation of the image exam. The report may even take a little longer. However, in the same way that not only the description of a nodule of an abdominal organ is important, but also the precise size and location, anatomical and characteristic correlations (flow and contrast enhancement) as they directly interfere in the diagnosis and management, the precise descriptions of abdominal hernias have the same impact mainly on surgical planning. 

At this moment, for the fastest and widest dissemination of this concept, it is essential that surgeons involve and encourage radiologists to adopt the routine performance of a structured report with the necessary information about AW. And we believe that the structured report proposal, presented in this article, can help radiologists in their work.
